# Retinoic acid modulation guides human-induced pluripotent stem cell differentiation towards left or right ventricle-like cardiomyocytes

**DOI:** 10.1186/s13287-024-03741-0

**Published:** 2024-06-21

**Authors:** Hengliang Zhang, Payel Sen, Jules Hamers, Theresa Sittig, Brent Woestenburg, Allessandra Moretti, Andreas Dendorfer, Daphne Merkus

**Affiliations:** 1 Walter Brendel Center for Experimental Medicine (WBex), University Clinic Munich, LMU Munich, 81377 Munich, Germany; 2 Center for Cardiovascular Research (DZHK), Munich Heart Alliance (MHA), Partner Site Munich, 81377 Munich, Germany; 3 Interfaculty Center for Endocrine and Cardiovascular Disease Network Modelling and Clinical Transfer (ICONLMU), LMU Munich, Munich, Germany; 4 Division of Experimental Cardiology, Dept of Cardiology, Erasmus University Medical Center, 3000CA, Rotterdam, The Netherlands; 5 First Department of Medicine, Klinikum Rechts der Isar, School of Medicine and Health, Technical University of Munich, Cardiology, Munich, Germany; 6 Regenerative Medicine in Cardiovascular Diseases, First Department of Medicine, Klinikum Rechts der Isar, School of Medicine and Health, Technical University of Munich, Munich, Germany; 7 The First Affiliated Hospital, College of Clinical Medicine of Henan, University of Science and Technology, Luoyang, China

**Keywords:** Retinoic acid, hiPSC, Cardiomyocyte, Engineered heart tissue, Left ventricle

## Abstract

**Background:**

Cardiomyocytes (CMs) derived from human induced pluripotent stem cells (hiPSCs) by traditional methods are a mix of atrial and ventricular CMs and many other non-cardiomyocyte cells. Retinoic acid (RA) plays an important role in regulation of the spatiotemporal development of the embryonic heart.

**Methods:**

CMs were derived from hiPSC (hi-PCS-CM) using different concentrations of RA (Control without RA, LRA with 0.05μM and HRA with 0.1 μM) between day 3-6 of the differentiation process. Engineered heart tissues (EHTs) were generated by assembling hiPSC-CM at high cell density in a low collagen hydrogel.

**Results:**

In the HRA group, hiPSC-CMs exhibited highest expression of contractile proteins MYH6, MYH7 and cTnT. The expression of TBX5, NKX2.5 and CORIN, which are marker genes for left ventricular CMs, was also the highest in the HRA group. In terms of EHT, the HRA group displayed the highest contraction force, the lowest beating frequency, and the highest sensitivity to hypoxia and isoprenaline, which means it was functionally more similar to the left ventricle. RNAsequencing revealed that the heightened contractility of EHT within the HRA group can be attributed to the promotion of augmented extracellular matrix strength by RA.

**Conclusion:**

By interfering with the differentiation process of hiPSC with a specific concentration of RA at a specific time, we were able to successfully induce CMs and EHTs with a phenotype similar to that of the left ventricle or right ventricle.

**Supplementary Information:**

The online version contains supplementary material available at 10.1186/s13287-024-03741-0.

## Background

Cardiomyocytes (CMs), derived from human induced pluripotent stem cells (hiPSC-CMs), and the engineered heart tissue (EHT) derived from these hiPSC-CMs, constitute a highly advantageous in vitro experimental model for conducting personalized drug screening and advancing regenerative strategies within the realm of precision medicine [27, 37]. However, CMs induced from hiPSCs using traditional methods represent a heterogeneous population comprising both atrial and ventricular cells [29, 64]. Although, earlier studies have effectively accomplished the differentiation of hiPSC-CM into distinct atrial or ventricular phenotypes [14, 30], no prior research has achieved the successful differentiation of hiPSC-CM into EHTs with specific phenotypes corresponding to either the left ventricle (LV) or the right ventricle (RV).

**Fig. 1:**
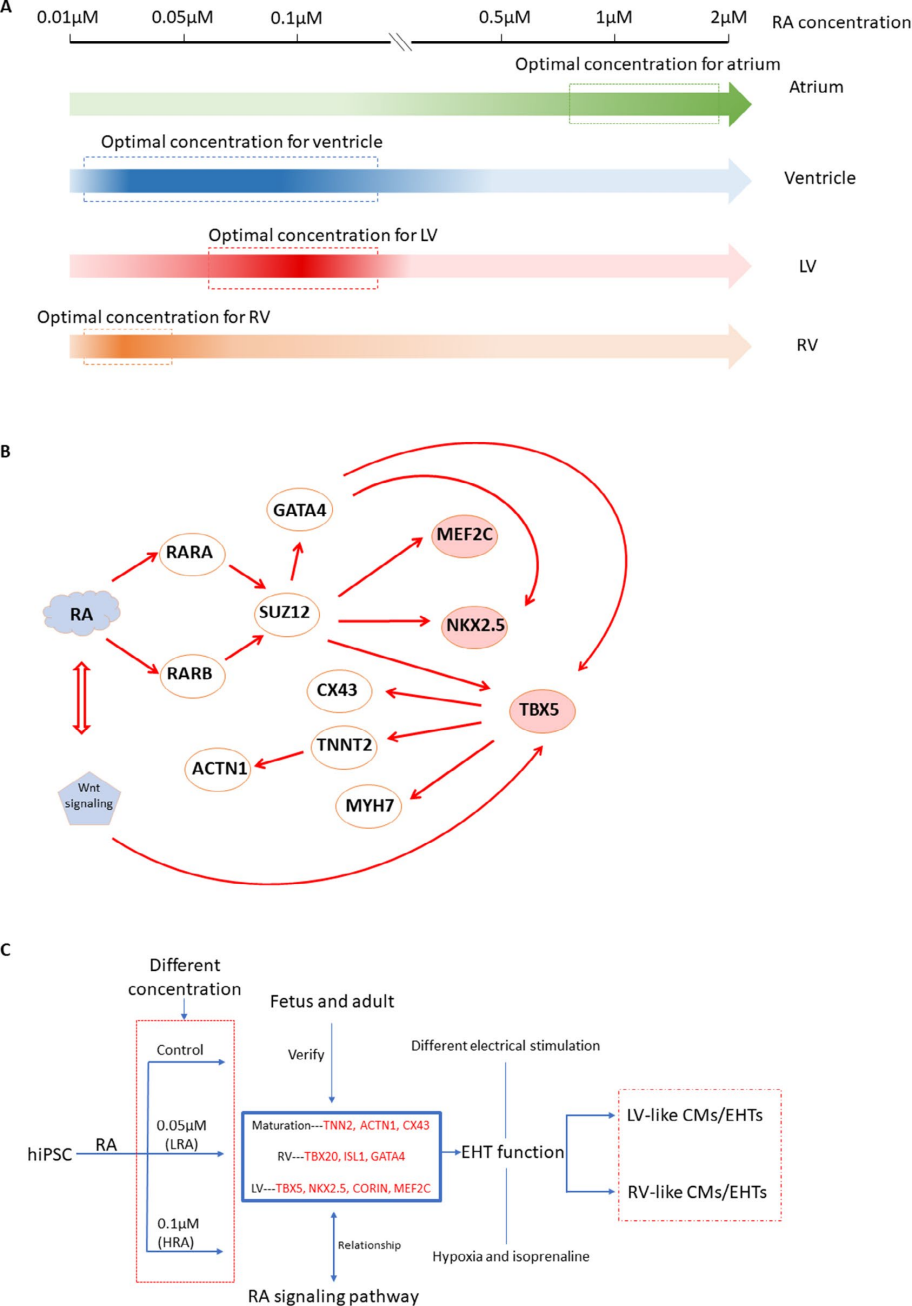
Retinoic acid (RA) signaling plays a pivotal role in iPSC differentiation. **A** RA regulates the differentiation direction of CMs in a concentration dependent manner. **B** RA regulates hiPSC-CM differentiation direction through left ventricular-specific transcription factors. **C** Schematic presentation of this study. Gene names with a red colour represent marker genes or/and transcription factor genes. hiPSC: human induced pluripotent stem cell. RA: Retinoic acid. LRA, low concentration RA—0.05 µM. HRA, high concentration RA—0.1 µM. LV: Left ventricle. RV: Right ventricle. EHT: Engineered heart tissue. CM: Cardiomyocyte

Retinoic acid (RA) signaling plays a pivotal role in embryonic development, as it is essential for organizing the trunk and facilitating organogenesis in diverse tissues derived from all three germ layers [16, 22]. In addition to being one of ingredients regulating embryonic development, RA also regulates cardiac development and affects the differentiation of hiPSC-CM into different subtypes, with the direction of differentiation being time- and concentration-dependent [59] (Fig. 1A). Previous research has demonstrated that RA concentrations ranging from 1 µM to 5 µM appear to promote hiPSC-CM or heart embryonic differentiation toward atrial CMs [14, 30, 59], whereas a concentration of 0.05 µM appears to promote differentiation towards left ventricular CMs [25]. Other studies found that RA mainly induces epicardial cells at 1 µM to 4 µM [23], while 0.5 µM to 1 µM of RA increases the proportion of atrial CMs [14, 51]. Not only RA concentration, but also the timing of RA intervention is critical, with recent in vivo and in vitro findings showing that low RA dosage at the mesoderm induction stage is critical for LV CM specification [15, 26, 29, 31].

Numerous studies have endeavoured to uncover the regulatory relationships among transcription factors associated with heart development. The transcription factors TBX5, NKX2.5, and GATA4 interact with each other to orchestrate the signal transduction pathways involved in heart development [6, 24, 34]. GATA4 activates the expression of NKX2-5, and both GATA4 and NKX2-5 activate the expression of TBX5 [5, 38, 57]. Additionally, the proteins NKX2.5 and MEF2C have been linked to ventricular CM differentiation [7, 33, 52]. TBX5 alone or in combination with GATA4, NKX2.5 and MEF2C regulates the expression of multiple target genes that are critical to the structure and function of the heart, including MYH6, MYH7 and NPPA [11, 65]. As RA has been shown to operate upstream of the transcription factor TBX5, indirectly modulating its expression [18, 61], RA concentrations may govern gene and protein expression crucially involved in heart morphogenesis (Fig. 1B). Hence, modulating RA concentrations may provide a tool to direct CM-differentiation towards LV and RV phenotypes.

Therefore, in the present study, we explored a new method of left and right ventricular CM and subsequent EHT generation while optimizing concentration and timing of RA intervention during hiPSC differentiation. Specifically, the first aim of our study was to investigate whether specific concentrations of RA can guide the hiPSCs differentiation towards LV and RV phenotypes. To achieve the goal, we intervened between the 3rd and 6th days of the differentiation process of hiPSC-CM with different concentrations of RA, according to previously published studies [14, 25]. To verify whether the differentiated hiPSC-CMs were LV and/or RV-like, we derived LV and RV specific marker genes from single-cell RNA sequencing data available in literature, and subsequently verified the function and specificity of these marker genes in explanted adult human heart tissue. The second aim of our study was to investigate whether the LV- and RV-like phenotypes of the iPSC-derived CMs were preserved in EHTs, which more closely mimic functional myocardial tissue. Thereto, the contractile force of EHTs was examined and compared, alongside their responses to hypoxia, β-receptor agonists, and electrical stimulation (Fig. 1C).

## Materials and methods

### Analysis of the fetal heart single-cell RNA sequencing dataset

The single-cell RNA sequencing dataset GSE106118 was acquired from GEO [13]. The dataset included human fetal right and left ventricular specimens obtained from aborted fetuses aged 5 to 24 weeks with appropriate informed consent and ethical approval. Differential gene expression analysis was performed using the FindMarkers function in Seurat, and genes with an adjusted *P*-value < 0.05 and a |log-fold change| > 2 were considered differentially expressed. Cell-clustering analyses were performed with the FindClusters function of the “Seurat” package with proper resolutions. The t-distributed stochastic neighbor embedding (t-SNE) was used to display identified cell clusters. The data were automatically annotated through HumanPrimaryCellAtlasData (https://rdrr.io/github/LTLA/celldex/man/HumanPrimaryCellAtlasData.html). In order to increase the accuracy of annotations, the annotation results were rechecked based on highly expressed genes, uniquely expressed genes, and reported canonical cellular markers.

### Verification of LV and RV marker genes

The marker genes for LV and RV were verified in human heart tissue. Transmural sections of left and right ventricular myocardium, measuring 2 × 2 cm^2^, were obtained from explanted human failing hearts. Upon retrieval, myocardial specimens were immediately placed in cold (4 °C) buffer (136 mM NaCl, 5.4 mM KCl, 1 mM MgCl2, 0.33 mM NaH2PO4, 10 mM glucose, 0.9 mM CaCl2, 30 mM 2,3-butadione-2-monoxime, 5 mM HEPES, pH 7.4) [19].

### Cell culture and differentiation of hiPSC-CMs

Pluripotent stem cells were obtained by reprogramming somatic cells from two healthy human donors (resulting in two clones of hiPSC) according to the prescribed methods [40]. Clone 1 was derived from skin fibroblasts in the laboratory of Dr Moretti, while clone 2 was derived from erythroid progenitors (commercially available). The study participant provided written informed consent and the investigation conformed to the principles outlined in the Declaration of Helsinki. For maintenance culture, hiPSCs were cultured in matrix-coated dishes (Geltrex LDEV-Free, Gibco), using Essential 8 medium (Gibco), which was changed on a daily basis. Cells were split at 85% confluency, using EDTA for dissociation (Versene solution; Gibco). A cutting-edge differentiation procedure for hiPSC-CMs that employs small molecules to stimulate differentiation was described by Chen and colleagues [10]. In brief, cardiac differentiation was triggered when the cells reached around 85% confluence by culturing cells for 24 h in Cardiomyocyte differentiation medium A which pushes hiPSCs toward mesodermal commitment via BMP/activin pathway activation and glycogen kinase 3 inhibition. Subsequently, Cardiomyocyte differentiation medium B (PSC Cardiomyocyte Differentiation Kit; Gibco), which induces cardiac mesoderm via Wnt inhibition, was added for 48 h. Cardiomyocyte maintenance medium (PSC Cardiomyocyte Differentiation Kit; Gibco) was then used for 12 days to further promote differentiation of hiPSC-CMs. Between day 3 and 6 of differentiation, RA (dissolved in DMSO) was introduced into cardiomyocyte maintenance medium (Figure S1A) in two concentrations: 0.05 µM (LRA, Low concentration retinoic acid) and 0.1 µM (HRA, High concentration retinoic acid), while the control group received the same concentration of DMSO as the other groups, but without RA. hiPSC-CMs began to beat spontaneously starting on the 7th or 8th day of the differentiation protocol. Hence, we recorded the beating frequency of each well in each group from day 8 onwards.

### Primary tissue assembly

Differentiated hiPSC-CMs were dissociated with TrypLE Select Enzyme (Gibco) for 8 min, followed by collagenase II (1.5 mg/mL, Sigma-Aldrich) for 7 min. Cells were dispersed in EB6 medium (Table S1) and centrifuged at 390 × g for 5 min. The cell pellet was suspended in EB6 medium to which 0.55 mg/mL bovine collagen I (Gibco), 0.08 mg/mL Geltrex (LDEV-Free, Gibco), and 1% RevitaCell supplement was added (modified from [9]), reaching a cell concentration of 1.1 × 10^5^ cells/µL. Subsequently, 55 µL of the cell-matrix mixture was pipetted on a 30 mm organotypic filter (PICMORG50, Merck Millipore) to create a disc approximately 8 mm in diameter and 2 mm in thickness. Following solidification of the tissue disc for 30 min at 37 °C, 1 mL of EB6 medium was introduced beneath the filter and replaced every other day for a total of 5 days in culture (Figure S1B).

### Biomimetic culture of EHT

Our team has devised a biomimetic cultivation system for the extended preservation of adult human myocardial slices, which allows precise modulation of preload and afterload, with continuous bipolar stimulation, and medium agitation [19]. This method has also been used for EHT maturation [36] as detailed below. The modified chambers used in this study were constructed using injection molded polystyrene blanks (Proto Labs, Feldkirchen, Germany). The EHTs were stimulated using bipolar, current-controlled electrical pulses with a duration of 2 × 1 ms, amplitude of 50 mA, and a frequency of 1 Hz (60 bpm). The culture system was maintained in a standard incubator (at 37 °C, 3% CO_2_, and 80% humidity) and connected to an external computer via USB, which ran custom software for stimulation control and data acquisition. Further analysis of the data was performed using LabChart Reader software (ADInstruments, Australia).

The primary EHTs were transferred from organotypic filters to biomimetic culture chambers (BMCCs, InVitroSys, Gräfelfing, Germany) pre-filled with 2.4 mL of modified EHT culture medium (adapted from [53], Table S2, pre-warmed to 37 °C). The EHTs were positioned in the BMCCs by puncturing their centers with adjacent holding posts, which were then adjusted to a distance of 3 mm to create a ring-shaped EHT that was immediately subjected to electrical stimulation (1 Hz, 50 mA current) and stretch conditioning. To promote EHT maturation and minimize the risk of damage (as demonstrated in our prior work [36]), the EHTs were distended once a day by manually moving the sliding fixation hook (adjustable post) at a rate of 0.16 mm/day for three days. The medium was partially exchanged every other day (with 1.6 mL of fresh medium), which included the addition of 0.1 nmol/L 3,3′,5-triiodo-L-thyronine (Sigma-Aldrich). Eleven independent experiments from two clones were conducted, and EHTs were cultured for 7 days. On the final day of cultivation, various parameters such as contractility and spontaneous beating frequency of EHT under distinct conditions were evaluated.

Systolic and diastolic forces (preload) of the EHTs were continuously assessed throughout the whole cultivation period and different stressors were introduced to assess their effect on contractile function of EHTs.

Response to increasing contraction frequency: Contraction frequency was altered by modulation of the frequency of electrical stimulation in EHTs. Under baseline conditions, EHTs were electrically stimulated at 1.0 Hz. In the 1.5 Hz group, electrical stimulation frequency was gradually increased from 1 Hz by 0.1 Hz per minute until the frequency of 1.5 Hz was reached. In the 3.0 Hz group, electrical stimulation frequency was gradually increased from 1.0 Hz by 0.2 Hz per minute until the frequency of 3.0 Hz was reached.

Hypoxia: For induction of tissue hypoxia, medium agitation was stopped for a 2-minute interval. Electrical stimulation was maintained at 1.0 Hz and contraction force recording was continued throughout.

Response to beta-adrenergic stimulation: Incremental concentrations of the non-selective beta-adrenoceptor agonist isoprenaline (10^− 9^ to 10^− 6^ M in Tyrode solution) were added into the BMCC in 2 h intervals. Contractile force was continuously recorded. Drug-induced changes in generated force, relaxation duration, and contraction duration were determined.

### Quantitative PCR analysis

RNA extraction of heart tissue, iPSC derived CMs and EHTs was carried out with the RNeasy Mini Kit (Qiagen). RNA integrity number (RIN) was determined using Simplinano Spectrophotometer (Biochrom). Equivalent amounts of RNA were reversely transcribed using the QuantiTect Reverse Transcription Kit (Qiagen), according to the manufacturer’s instructions. All qRT-PCR analyses were conducted in duplicate on a StepOnePlus Real-Time PCR System (Applied Biosystems), with the following cycling conditions: a holding stage at 95 °C for 10 min, followed by 40 cycles at 95 °C for 15 s, 60 °C for 1 min, and 72 °C for 15 s. The gene expression results were analyzed using the 2^^−ΔΔCT^ method, and glyceraldehyde-3-phosphate dehydrogenase (GAPDH) was used as an endogenous control for mRNA expression. The primer list is available in table S3.

### RNA sequencing of EHTs

Messenger RNA was purified from total RNA of 3 EHTs per group using poly-T oligo-attached magnetic beads. After fragmentation, the first strand cDNA was synthesized using random hexamer primers, followed by the second strand cDNA synthesis using either dUTP for directional library or dTTP for non-directional library. The library was checked with Qubit and real-time PCR for quantification and bioanalyzer for size distribution detection. Quantified libraries were pooled and sequenced on Illumina platforms, according to effective library concentration and data amount. Raw data (raw reads) of fastq format were first processed through in-house perl scripts. FeatureCounts [32] v1.5.0-p3 was used to count the reads numbers mapped to each gene. Then FPKM of each gene was calculated based on the length of the gene and reads count mapped to this gene. Differential expression [2] analysis was performed using the DESeq2Rpackage (1.20.0). Gene Ontology [62] (GO) enrichment analysis of differentially expressed genes was implemented by the cluster Profiler R package. GO terms with corrected *P*-value < 0.05 were considered significantly enriched by differential expressed genes.

### Immunofluorescence staining

After being washed with PBS three times, whole cells or tissues were prepared for fixation. Cells and EHTs were placed in 1 mL of a 30% sucrose solution in PBS at 4 °C overnight. Subsequently, cells and EHTs were washed for 30 min with PBS, permeabilized for 60 min with 1% Triton X-100 in PBS and incubated for 1 h in blocking solution (3% bovine serum albumin and 1% fetal calf serum in PBS). The primary monoclonal anti-α-actinin antibody (sarcomeric, 1:100, Sigma-Aldrich) was added in antibody dilution buffer overnight at 4 °C. On the next day, samples were incubated with goat anti-mouse IgG (H + L) and a highly cross-adsorbed secondary antibody, Alexa Fluor 546 (Invitrogen), at 1:100 in antibody dilution buffer for 2 h. Subsequently, cells and tissues were washed with PBS three times for 10 min and incubated overnight at 4 °C with DAPI (2 µmol/L, Invitrogen).

### Quantitation and statistical analysis

Data are shown as the mean +/- standard error of the mean (SEM). One-way ANOVA and Student’s t test were used to test for treatment effects, as appropriate. All statistical analyses were performed with GraphPad Prism 7. Statistical significance was accepted at an error level of *P* < 0.05. The number of independent experiments performed for each data set was detailed in the figure legends.

## Results

### RA signaling and determination of left and right ventricular marker genes in fetal heart

**Fig. 2:**
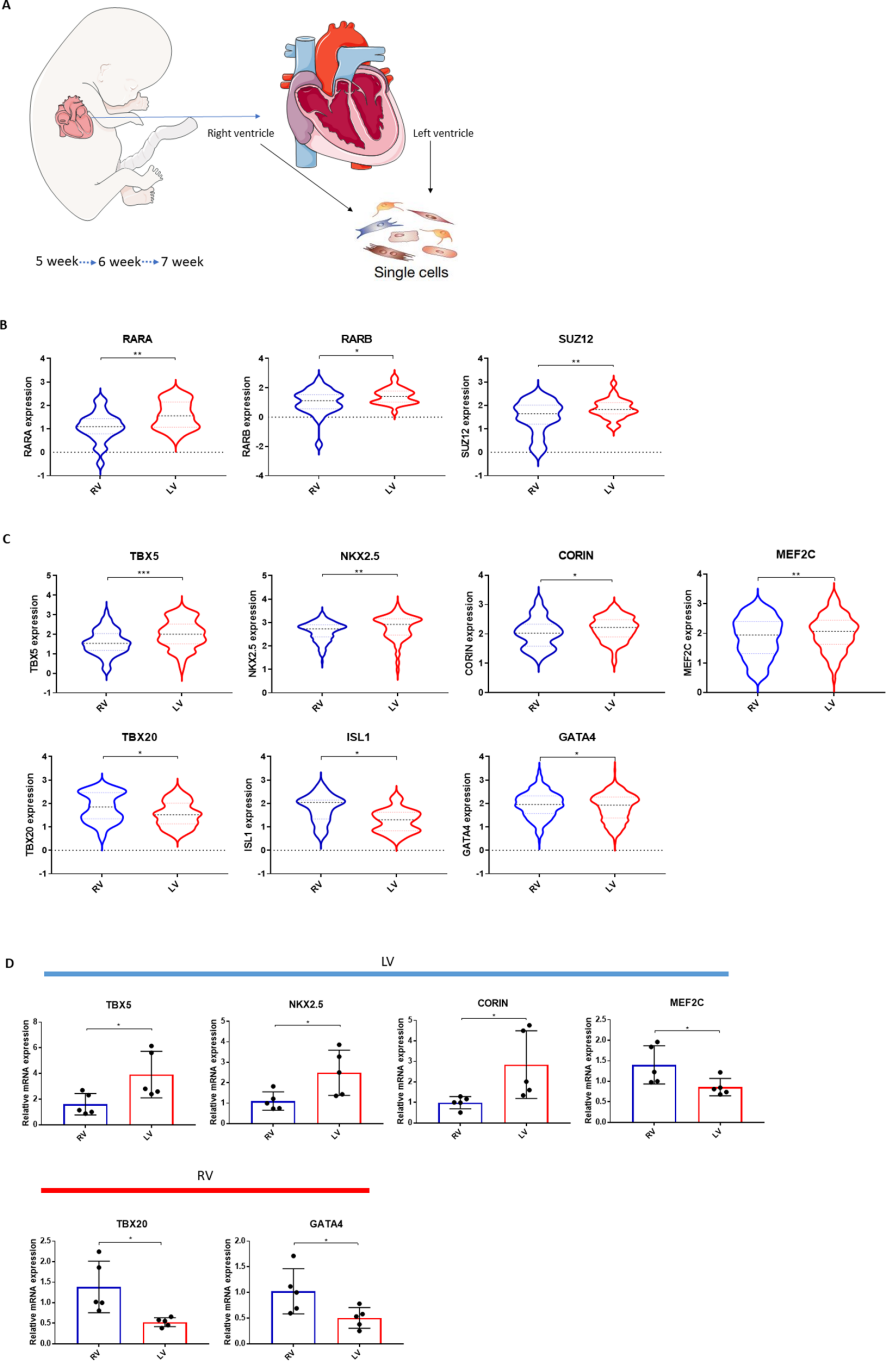
RA signaling and determination of left and right ventricular marker genes. **A** Introduction to the GSE106118 single cell RNA sequencing dataset (left). MYH6 and MYH7 gene expression in fetal LV and RV CMs (right). **B** The genes involved in the RA signalling pathway shows high expression in left ventricular CMs. **C** The marker genes exhibited different expression between left and right ventricular CMs. B and C: Unpaired t-test between LV and RV. *p < 0.05, **p < 0.05, ***p < 0.001. Samples in which gene expression was 0 in the original data were excluded, and all values were converted by log10. **D** Expression of marker genes in adult humans. Unpaired t-test between RV and LV. *p < 0.05, **p < 0.01

In order to determine a potential role for RA signaling in LV/RV differentiation, single cell RNA sequencing data from fetuses aged 5–7 weeks (start of cardiac contraction) were extracted from the GSE106118 dataset (Fig. 2A) and further analyzed, separating the LV dataset and RV datasets. Principal component analysis combined with t-distributed stochastic neighbor embedding (t-SNE) showed the distribution of various cell types in the LV and RV being noticeably different (Figures S2A, S2B and S2C). Expression of the contractile proteins MYH6 and MYH7 was higher in fetal LV CMs as compared to the fetal RV CMs (Fig. 2A). Consistent with a role for RA signalling in differentiation into LV versus RV, expression of genes involved in RA signaling (RARA, RARB and the polycomb repressor 2 subunit SUZ12) was higher in fetal CMs of the LV as compared to the RV (Fig. 2B).

SUZ12 is known to regulate expression of transcription factors in embryonic development [28, 35], hence, expression of transcription factors and enhancers (TBX5, TBX20, NKX2.5, MEF2C, GATA4, ISL1) was determined within the LV and RV CM populations. TBX5 and NKX2.5 revealed a significantly higher expression in LV CMs as compared to RV CMs, with very limited expression in other cell types whereas GATA4, TBX20 and ISL1 were higher expressed in the RV CMs as compared to the LV CMs. Expression of MEF2C was exhibited higher in the LV CMs compare to the RV CMs (Fig. 2C). Expression of the cardiospecific enzyme CORIN was higher in the LV CMs as compared to the RV CMs. Together with previous studies [50, 64], our data suggest that high expression of TBX5, NKX2.5 and CORIN can be used as markers of left ventricular CMs, whereas TBX20, ISL1 and GATA4 can be used to delineate RV CMs.

### Validation of marker genes in adult human hearts

Expression levels of the above mentioned genes as marker genes for LV and RV was further confirmed in adult human LV and RV. TBX5, NKX2.5 and CORIN showed a higher expression in human LV as compared to RV, while GATA4 and TBX20 showed a higher expression in RV as compared to LV (Fig. 2D). Surprisingly, MEF2C has a higher expression in human adult RV, although this may be part of the RV hypertrophic and/or stress response due to LV failure [12]. ISL1, as a marker gene for right ventricular progenitor cells, was not expressed in adult hearts.

### Effect of RA on hiPSC-CM maturation and differentiation

**Fig. 3:**
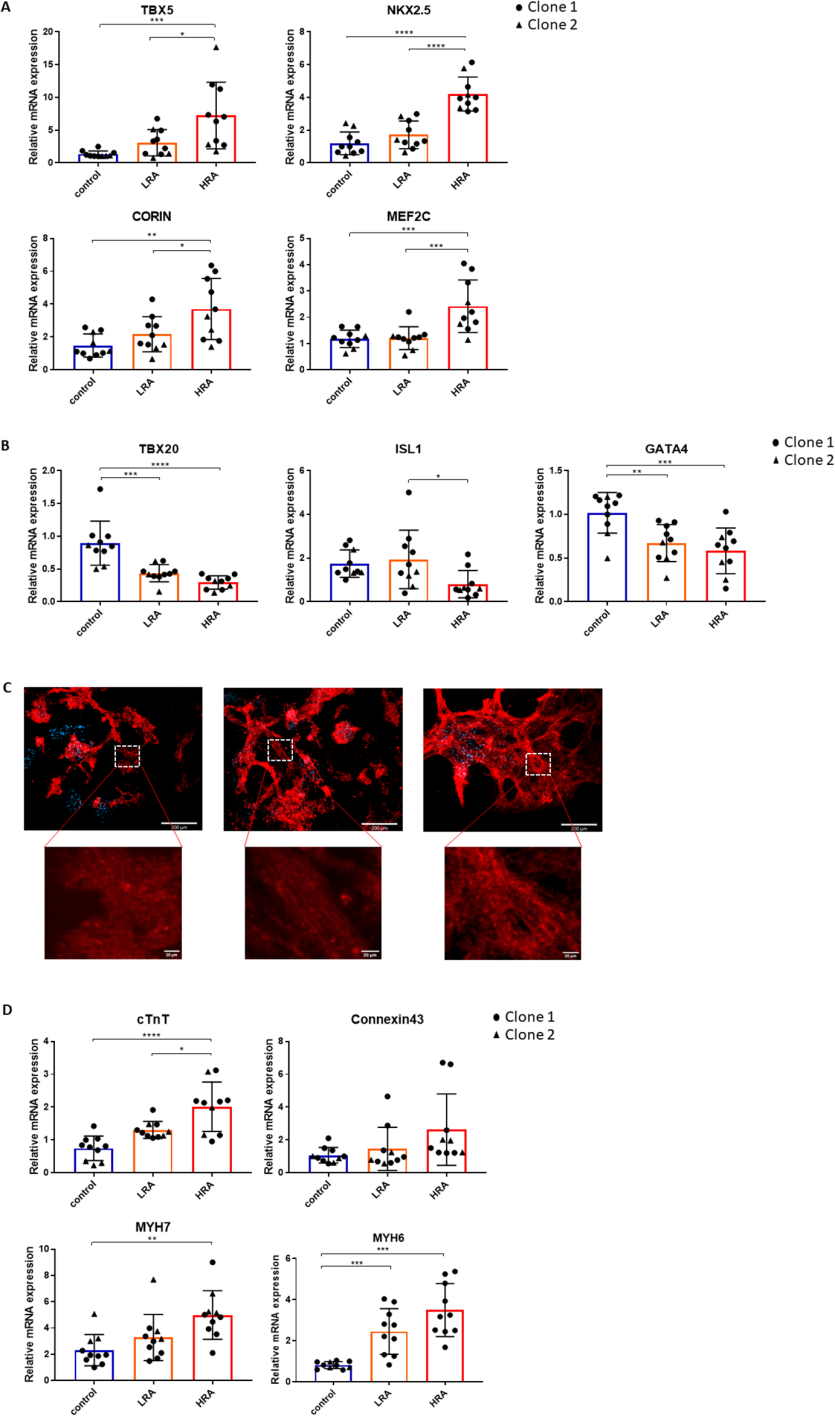
Effect of RA on hiPSC-CM maturation and differentiation. **A** Left ventricular marker genes expressed in hiPSC-CMs. **B** Right ventricular marker genes expressed in hiPSC-CMs. **C** α-actinin expression in hiPSC-CM. Left: control. Middle: LRA. Right: HRA. Immunofluorescent images of hiPSC-CM cultured for 26 days. Red: α-actinin. Blue: DNA. Scale bar: 200 μm. **D** Relative mRNA expression of marker genes of CM maturation. **A**, **B** and **D**: One-way ANOVA, Tukey’s multiple comparison test. *p < 0.05, **p < 0.01, ***p < 0.001, ****p < 0.0001

Consistent with previous studies, iPSC culture and differentiation resulted in a network of CM monolayers (Figure S1B), that started to beat spontaneously at day 6–9 of differentiation. Expression of transcription factors TBX5, NKX2.5, MEF2C and the enzyme CORIN was highest in the HRA group (Fig. 3A). Conversely, expression of TBX20 and GATA4 was highest in the control group, whereas ISL1 showed the highest expression in LRA group (Fig. 3B). These data suggest that RA in a concentration of 0.1µM promoted differentiation towards a LV phenotype, whereas RA concentrations ≤ 0.05 µM promoted differentiation towards an RV like phenotype. Although overall morphology of the hiPSC-CM was not different between groups, RA promoted maturation of the iPSC-derived CMs, with more organized α-actinin expression in the HRA group (Fig. 3C). Furthermore, higher mRNA expression of cTnT, MYH6 and MYH7 was observed in the HRA group as compared to control, whereas expression of connexin 43 was not different (Fig. 3D) at 26 days of differentiation and maturation.

The LV primarily derives from the FHF, whereas the RV originates from the SHF. Within the HRA group, a high expression of the FHF marker gene, THBS4, was observed, along with notable expression of HCN4. Contrarily, the SHF marker gene, WNT5A, exhibited high expression within the control group (Figure S3A). The marker gene of ventricle, MLC2V, exhibited high expression in HRA group (Figure S3B).

### Effect of RA on early iPSC differentiation persists in EHTs: RA improves EHT maturity through altered gene-expression related to cell-cell and cell-matrix interaction but not ion channels and metabolism

**Fig. 4:**
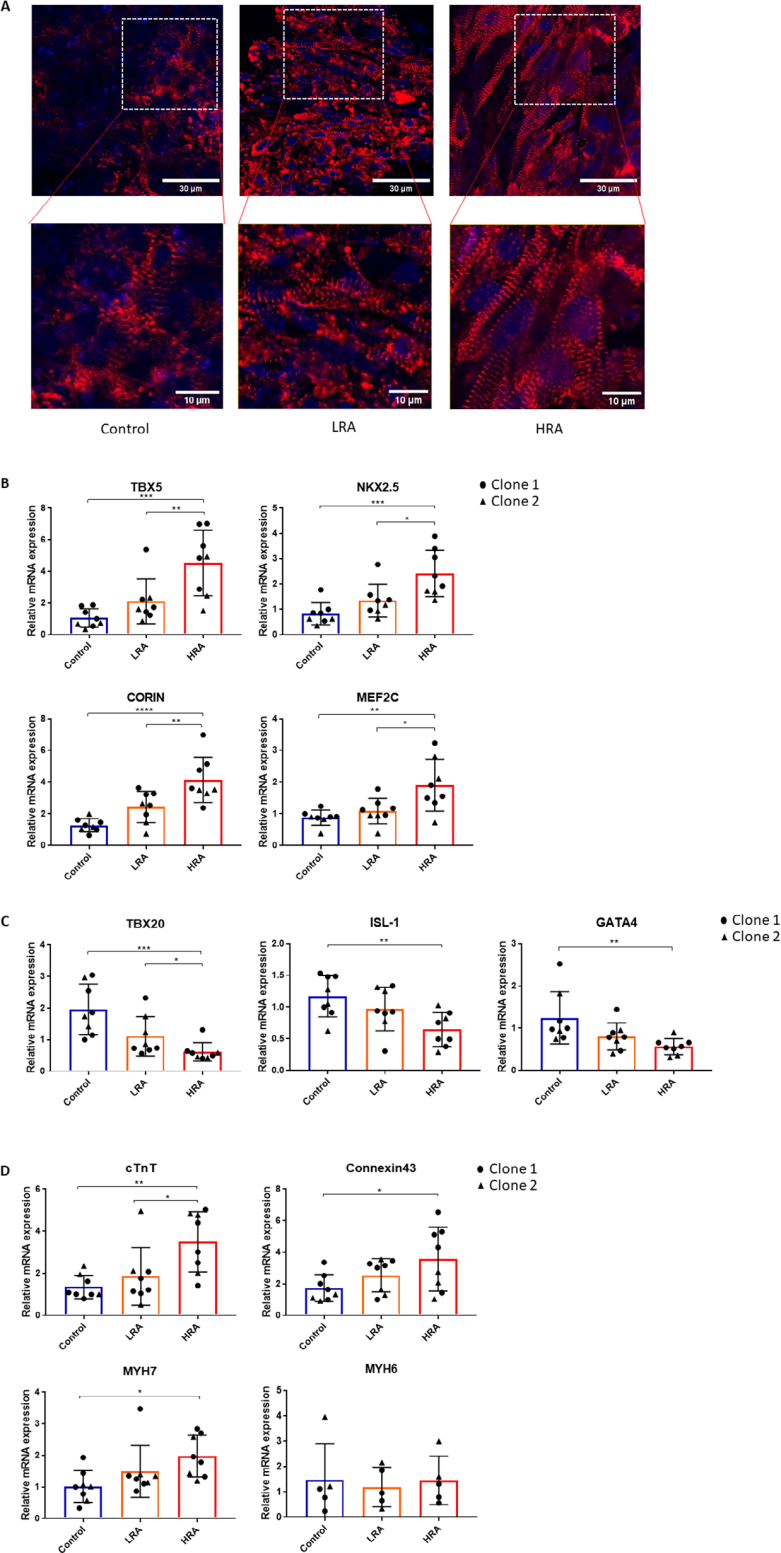
Effect of RA on early iPSC differentiation persists in EHTs. **A** Immunofluorescence images of EHT across various experimental groups are presented. Upper Panel: The spatial distribution of α-actinin exhibits variability across distinct EHT groups. Red: α-actinin. Blue: DNA. Scale bar = 30 μm. Lower Panel: A localized magnification reveals variations in sarcomere quantity and quality within the different experimental groups. Red: α-actinin. Blue: DNA. Scale bar = 10 μm. **B** Marker genes of LV expressed in different groups of EHT. **C** Marker genes of RV expressed in different groups of EHT. **D** Marker genes of CM maturation expressed in different groups of EHT. **B**-**D**: One-way ANOVA, Tukey’s multiple comparison test. *p < 0.05, **p < 0.01, ***p < 0.001, ****p < 0.0001

iPSC-derived CMs were further matured into EHTs, to assess whether the differences induced by RA persisted in this maturation step. Immunofluorescence staining revealed a high expression and organization of α-actinin within the HRA-EHTs, leading to enhanced sarcomere visibility (Fig. 4A).

Consistent with the gene-expression in the iPSC-derived CMs, expression of TBX5, CORIN, MEF2C and NKX2.5 were highest (Fig. 4B), while TBX20, GATA4 and ISL1 expression was lower in HRA-EHTs, as compared to control-EHTs (Fig. 4C), after 7 days of maturation in the BMCC. Furthermore, markers of maturation and contractile function, cTnT, MYH7 and connexin43 were highest in HRA-EHTs, while MYH6 was not different (Fig. 4D).

To further investigate molecular changes underlying the improved organisation of the EHTs from CMs previously exposed to RA, RNA sequencing was performed. We detected 101 upregulated and 679 genes downregulated (*p*-value < 0.05 and fold change ≥ 1.3) in the HRA-EHTs vs. control EHTs respectively.

**Fig. 5:**
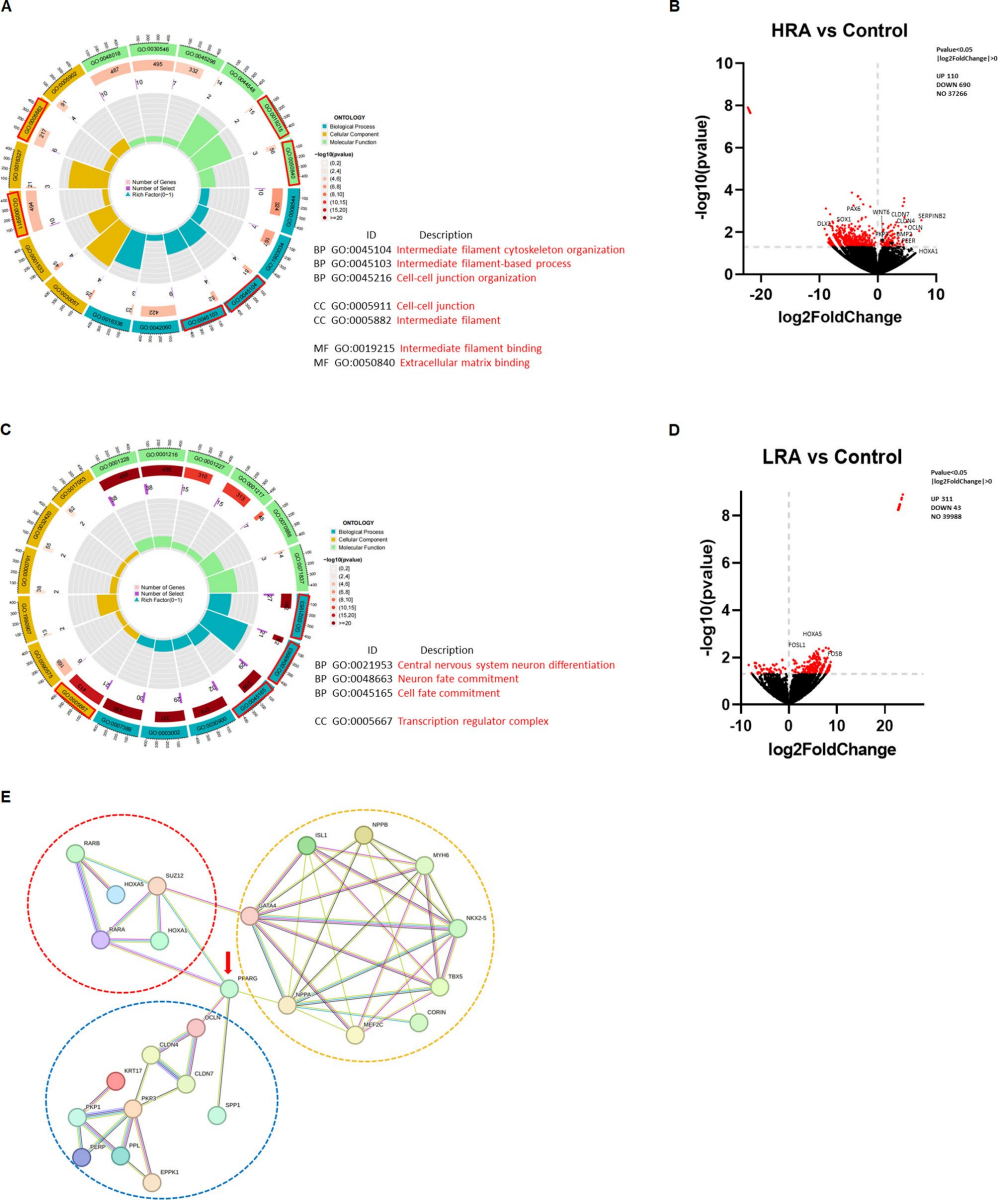
The extracellular matrix plays a crucial role in contributing to the contractile force of EHT. **A** GO terms illustrating the functional enrichment of upregulated DEGs between the HRA and control group. **B** Volcano plot displaying DEGs between HRA and control group. **C** GO terms illustrating the functional enrichment of down regulated DEGs between the HRA and control groups. **D** Volcano plot displaying DEGs between LRA and control group. **E** Protein-protein interaction network illustrating the relationship between RA signaling pathway, transcription factors and the extracellular matrix. The red circle represents proteins relevant to the RA signaling pathway. The yellow circle represents transcription factors relevant to ventricular differentiation. The blue circle represents proteins related to the extracellular matrix

GO enrichment pathway analysis showed upregulation of several genes coding for ECM remodelling (SerpinB2, F3, TGFa), intermediate filaments and cell-cell junctions (CLD7, OCLN, PPL) (Fig. 5A and B, Table S4). Volcano plot analysis detected increased expression for several genes which play a vital role in cardiogenesis (Wnt6, BMP2, HOXA1) (Fig. 5B) [59]. Furthermore, the significantly downregulated genes were primarily linked to the regulation of non-cardiomyocyte differentiation (Fig. 5C, Table S5). Expression of Na^+^, K^+^ and Ca^2+^-channels (Figure S4A) and glucose and fatty acid transporters (Figure S4B) was not significantly different between control EHTs and HRA-EHTs.

In the LRA vs. Con EHT, 311 were upregulated and 43 genes downregulated (*p*-value < 0.05 and fold change ≥ 1.3) in the LRA-EHTs respectively. The GO enrichment pathway analysis did not reveal any significant pathway-changes between these two groups. However, we did observe upregulation of transcription factors (FOSl1, FOSB, HOXA5) using a volcano plot analysis (Fig. 5D). Thus, both the comparisons (HRA vs. Con and LRA vs. Con) identified that RA treatment affected expression of HOX genes. This is relevant because HOX genes play a critical role during heart development [47] and several of these HOX genes feature RA-response elements (RAREs) in their enhancers or proximal promoters [1].

Finally, to elucidate the connection between RA pathway and intermediate filaments, extracellular matrix genes and HOX genes, a protein-protein interaction network analysis was conducted in order to provide additional insight into the RA signalling combining our RNA sequencing data and published data (Fig. 5E). The network reveals PPARG as the central hub gene. Numerous groups have presented PPARG as a critical factor in cardiac development but its specific role in inducing left versus right ventricle warrants further studies [43, 66].

### Effect of RA on EHT function

**Fig. 6:**
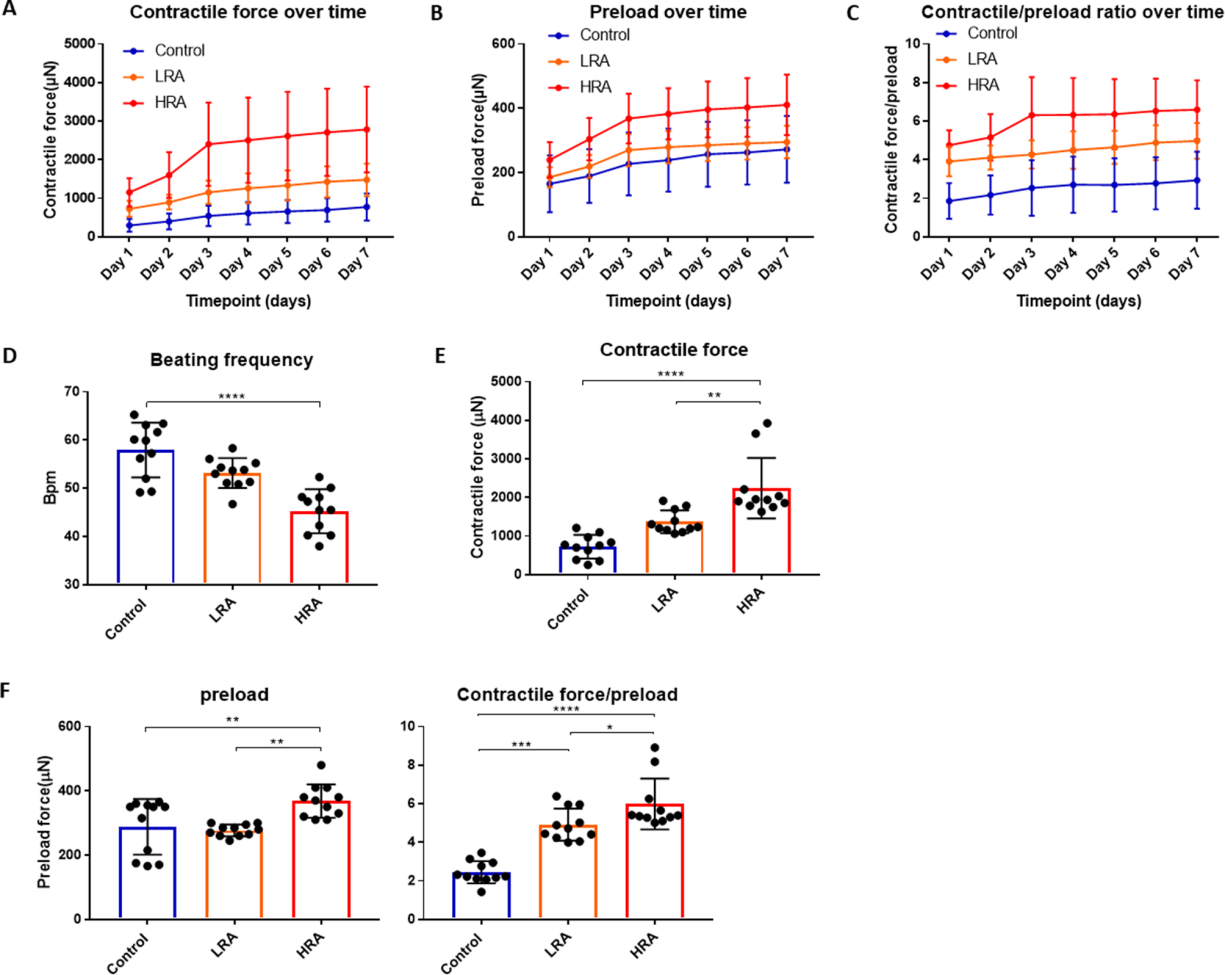
Effect of RA on EHT function. **(A)** The contractile force of EHT over time. *N* = 11. **(B)** The preload force of EHT over time. *N* = 11. **(C)** The contractile force/preload of EHT over time. *N* = 11. **(D)** The beating frequency of EHTs on fourth day. **(E)** The contractile force of EHTs on fourth day. **(F)** The preload force and contractile force/preload on fourth day. **D**-**F**: One-way ANOVA, Tukey’s multiple comparison test. *p < 0.05, **p < 0.01, ***p < 0.001, ****p < 0.0001

As EHT matured in BMCC, contractile force gradually increased over time in all groups, especially during the first three days when the preload was gradually increased (Fig. 6A&B). Even when corrected for the increased preload using the contractility index (force/preload), the increased contractile force was evident (Fig. 6C). On the fourth day in the BMCC, the supplemental preloading was discontinued, and the evaluation of EHT functionality among various groups commenced. Beating frequency was lower and contraction force was consistently higher in HRA-EHTs as compared to the LRA-EHTs and control-EHTs (Fig. 6D&E), even after correction for a higher preload, using the contractility index (force/preload) (Fig. 6F). The differences in gene expression induced by RA during hiPSC-CM differentiation translated into functional changes, that were persistently present.

### Response to stress

#### Hypoxia

**Fig. 7:**
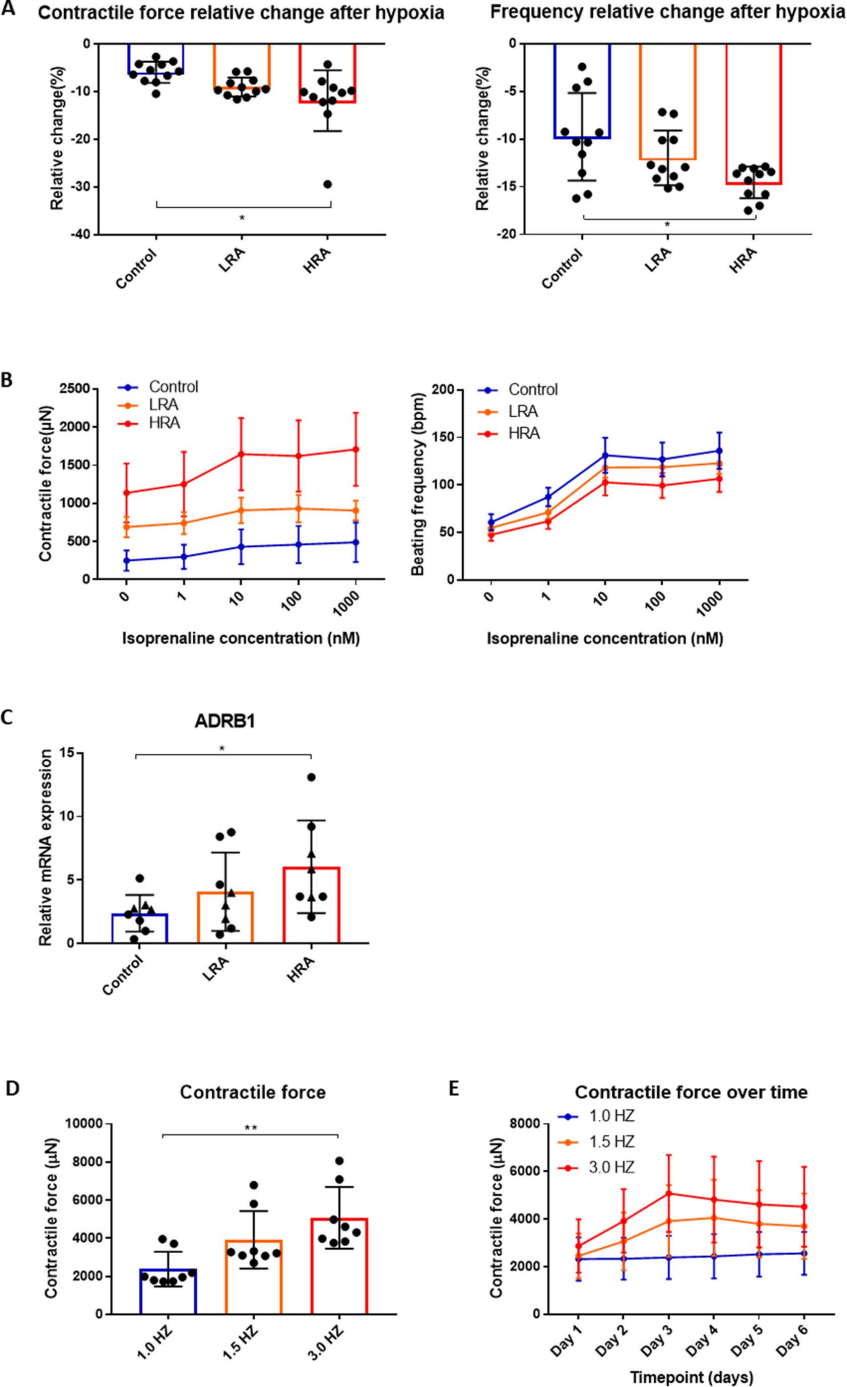
EHT response to stress. **(A)** The relative change of contractile force and beating frequency after hypoxia in different groups. **(B)** The response of EHTs to different concentrations of isoproterenol (Left). *N* = 11. **(C)** β-adrenoceptor gene expressed in different groups of EHT. **(D)** The contractile force at different electrical stimulation frequencies. **(E)** The contractile force at different electrical stimulation frequencies over time. *N* = 8. **A**, **C** and **D**: One-way ANOVA, Tukey’s multiple comparison test. *p < 0.05, **p < 0.01

The response of the EHTs to hypoxia was assessed by stopping the rocking of the BMCC system. The contractile force and beating frequency in HRA-EHTs were more sensitive to hypoxia as compared to the control-EHTs, while the LRA-EHTs showed an intermediate response (Fig. 7A). These data are consistent with HRA-EHTs showing a more LV-like phenotype, as the LV has a stronger contractile force and higher oxygen consumption compared to RV [3, 48].

#### *β-adrenergic stimulation*

The β-adrenoceptor agonist isoprenaline significantly increased the contractile force and beating frequency of the EHTs. The effect of β-adrenergic stimulation was most pronounced in the HRA-EHTs (Fig. 7B). RT-qPCR analysis revealed higher expression of the β_1_-adrenoceptor gene, ADRB1, within the HRA-EHT (Fig. 7C) as compared to Control, consistent with observations that expression of β_1_-adrenoceptors is highest in the adult LV ([3] and figure S5A). Interestingly, ADRB1 expression was not different in the iPSC-CMs (Figure S5B), suggesting that ADRB1 expression differences are only observed in more mature tissue.

Altogether, these data confirm a LV-like phenotype of HRA-EHTs and a RV-like phenotype of EHTs from the LRA and control groups.

#### High-frequency electrical stimulation

It has been proposed that increasing the intensity and frequency of electrical stimulation of the EHTs may promote tissue maturation and increase contractile force [46]. The frequency of electrical stimulation of EHTs was therefore gradually increased at day 7 after culture in BMCCs. For control-EHTs, stimulation at higher frequencies damaged the EHTs to such extent that measurements were not possible. For HRA-EHTs, contractile force after 72 h of electrical stimulation at 1.5 and 3.0 Hz was higher as compared to stimulation at 1.0 Hz, with 3.0 Hz yielding higher contractile force as compared to 1.5 Hz (Fig. 7D). Although there was a slight decrease in contractile force after switching to 1.0 Hz electrical stimulation, contractile force was still significantly higher than before applying electrical stimulation (Fig. 7E).

## Discussion

In our study, reanalysis of existing single cell sequencing dataset of fetal hearts yielded the characteristics of CMs in 5–7 week fetuses and screened out highly enriched genes of LV (TBX5, NKX2.5 and CORIN) and RV (TBX20 and ISL1) as potential markers of LV and RV development, that were confirmed in human adult (failing) myocardium, and subsequently used to classify hiPSC derived CMs as LV or RV like. We showed that addition of RA in a concentration of 0.1 µM (HRA group) resulted in a LV-like phenotype whereas absence of RA, or RA at a concentration of 0.05 µM (LRA group) resulted in a more RV-like phenotype. iPSCs-derived CMs were subsequently used to generate EHTs. HRA-EHTs showed higher expression of LV-enriched marker genes, higher contractile force and increased sensitivity to β-adrenergic stimulation and hypoxia, whereas control- and LRA-EHTs showed higher expression of RV-marker genes. The implication of our findings will be further discussed below.

Being able to control the differentiation of hiPSCs into distinct subtypes of CMs is essential for creating in vitro models of specific cardiovascular diseases and developing innovative treatments [8]. While previous studies have been largely successful, they have often utilized mixed populations of cardiovascular cells, including left ventricular-like cells, as well as small quantities of right ventricular, atrial, and pacemaker-like cells [4, 14, 20]. This approach presents a challenge, as the presence of heterogeneous cell types will significantly impact the outcomes of disease modeling in vitro. To accurately model and treat diseases that impact specific areas of the heart, it is imperative to develop precise differentiation strategies that can generate the specific CMs that belong to the LV or RV. Our results, together with results from previous studies [25, 63] suggest that RA regulates the differentiation of hiPSC into different CM subtypes in a concentration- and time dependent manner. A concentration of RA of 0.1 µM, promoted differentiation of hiPSC towards the left ventricular CMs, while concentrations of RA below 0.05 µM, promoted differentiation of hiPSC towards the right ventricular CMs. With a concentration of RA above 1µM, atrial CMs predominated over ventricular CMs [59].

Our study focused on the differentiation of hiPSCs into left and right ventricular lineages, and we have found that a precise concentration and timing of RA was necessary for left ventricular specification. The RA intervention phase aligned with the developmental stage of heart mesoderm cells and heart precursor cells in our study. Notably, the left ventricular marker genes, TBX5, NKX2.5, and CORIN, exhibited significantly increased expression in the HRA group after 20 days of differentiation. This discovery underscores the critical role of RA in driving hiPSC-CM differentiation towards a left ventricle-like phenotype. However, the precise mechanism through which RA signaling influences cardiac progenitor populations during hiPSC differentiation remains incompletely understood [17]. RA, derived from vitamin A, acts as a lipophilic molecule and serves as a ligand for nuclear RA receptors (RARs) [45], converting them from transcriptional repressors to activators. RA’s impact on development and cellular differentiation is mediated through retinoid receptors, which bind to specific DNA sequences called retinoic acid response elements (RAREs), thereby influencing the transcription of target genes [60].

Although the precise methodology through which RA guides hiPSC-CM differentiation towards a left ventricle-like phenotype remains unclear, our RNA sequencing results identified that RA treatment affected expression of HOX genes in EHTs, which is consistent with observations that HOX genes feature RA-response elements (RAREs) in their enhancers or proximal promoters [1]. As HOX genes have been shown to play a critical role during heart development [47], modulation of HOX may be a potential mechanism by which RA promotes hiPSC-CM differentiation. Furthermore, RA treatment was shown to upregulate expression of the transcription factors TBX5, NKX2.5 and MEF2C, while downregulating TBX20 and GATA4 in iPSC-CM as well as EHTs. These findings are consistent with their expression pattern in the LV and RV in the embryonic heart, and together with the specific ventricular expression pattern of the RA receptors RARA and RARB, provide a mechanism through which CMs are directed towards an LV lineage.

The use of primary human CMs derived from patients in in vitro studies poses challenges due to difficulties in their acquisition, preservation, and limited availability, which constrains their broader applicability in experimental research [56]. By combining different matrices or scaffolds with different cell types to simulate multicellular heart tissue, EHT is created. This product of the marriage of biology and tissue engineering can facilitate the understanding of extremely complex human (patho)physiology [42]. The BMCC system used in our study to cultivate EHT is one of the most advanced myocardial biomimetic culture systems. It provided a stable growth environment, nutrient supply, and allowed us to assess EHT functionality by manipulating environmental parameters during maturation [58]. The human LV and RV exhibit significant functional and structural differences. The LV has greater contractility, higher oxygen consumption/ increased sensitivity to hypoxia and pressure loads, but weaker automaticity compared to the RV [44]. Previous studies have found differences in ion channel mRNA expression as well as ion channel function between the left and right ventricles. Thus, NaV1.5 (SCN5A) mRNA expression, but not function was lower in RV than LV, and KCNQ1 mRNA expression and function was higher in RV as compared to LV, while other ion channels (KCNH2 (HERG) and CACNA1C (Cav1.2)) were not different [39]. Our RNA sequencing results did not reveal significant differences in mRNA expression of these ion channels between control EHTs and HRA-EHTs, although, consistent with literature, KCNQ1 appeared higher in control, RV-like EHTs as compared to HRA-EHTs. Furthermore, EHTs from the HRA group displayed higher expression of MYH7, but not MYH6, encoding for MHC-β and MHC-α respectively. Although MHC-α is considered the atrial MHC, it is also highly expressed in the ventricles during fetal development, and its expression may therefore represent a fetal rather than an atrial CM phenotype. The higher expression of MYH7 was accompanied by enhanced contractility, increased oxygen consumption (sensitivity to hypoxia), reduced automaticity, and heightened sensitivity to β-adrenergic stimulation. These findings, coupled with the high expression of left ventricular marker genes in the HRA group, collectively indicate that the left ventricular characteristics of iPSC-CM persist during EHT development, thereby providing an in vitro model for the future study of the LV, particularly in disease modeling, drug screening, precision medicine, and potentially even regenerative medicine [49].

Our study shows that RA treatment during differentiation modulates the contractile force of the final EHT by influencing expression of genes involved in the extracellular matrix, cell-cell junctions and intermediate filament proteins. Sequencing and functional data underscore the importance of cellular structural components, cell-cell and cell-matrix connectivity for development of cardiac force. The mRNA sequencing results and PPI network analysis suggest that PPARG may play a crucial role in regulating the extracellular matrix of EHT in the context of RA. This is consistent with data demonstrating that PPARG is important for myocardial development and ventricular septation [66].

Previous research has established that intermediate filament proteins, which are a key component of the cytoskeleton and conduct mechanical signals to the nucleus, can directly influence transcription of transcription factors such as NKX2.5, MEF2C thereby exerting an impact on myocardial regeneration and differentiation [41], which is in accordance with our data showing differential expression of these transcription factors in control EHTs vs. HRA-EHTs. In addition, EHTs derived from patients suffering from dilated cardiomyopathy showed that the absence of intermediate filament proteins resulted in myocardial tissue dilation, mitochondrial dysfunction, and diminished contractile capabilities [55]. Our research results are in concordance with previous investigations [21, 54], emphasizing that the generation of contractile force by EHTs is governed not only by CMs contractile function, but also by CM-CM and CM- extracellular matrix-connections that may subsequently impact the expression and function of transcription factors.

Altogether, our data highlight the structural and functional resemblance between EHTs and myocardial tissue. We successfully induced hiPSC differentiation into CM and EHT exhibiting left and right ventricle-like characteristics. Future studies are required to further finetune the RA concentrations and identify the exact differentiation pathways by which RA leads to left and right ventricular, as well as atrial CM lineages.

### Conclusion and future perspective

Intervening with RA early in the differentiation process of iPSCs can guide differentiation towards an LV or RV-like phenotype of iPSC-CMs. This phenotype is preserved during subsequent maturation in EHTs. The functional and genetic analysis of iPSC-CM and EHTs has provided insights into how the RA signaling pathway exerts its intricate control over cardiac development. Its activation of pivotal transcription factors that play a fundamental role in CM development, likely serve as the molecular mechanism behind RA’s facilitation of hiPSC differentiation into left ventricular CMs and subsequent maturation of LV-like EHTs. Indeed, the HRA group exhibited significantly elevated expression levels of CM maturation markers, as well as left ventricular markers, while the LRA and control groups showed more pronounced expression of right ventricular markers. Moreover, the HRA-EHTs demonstrated the highest contractility and the highest expression of the contractile protein and MYH7. Finally, our study substantiated the indispensable role of the extracellular matrix in augmenting the contractile force of EHTs.

These collective findings not only deepen our comprehension of cardiac differentiation but also establish a foundation for future investigations into in vitro left and right ventricular function, personalized drug screening, and the advancement of precision medicine.

### Electronic supplementary material

Below is the link to the electronic supplementary material.

Supplementary Material 1

Supplementary Material 2

Supplementary Material 3

## Data Availability

All data generated or analyzed during this study are included in this published article and its supplementary information files. The sequencing data of EHT were deposited into the Gene Expression Omnibus (GEO) database under accession number GSE245954.

## Abbreviations

BMCC: Biomimetic cultivation chamber
BMP: Bone morphogenetic protein
CM: Cardiomyocyte
CORIN: Corin, serine peptidase
CPC: Cardiac progenitor cell
DMEM: Dulbecco’s Modified Eagle Medium
DMSO: Dimethyl sulfoxide
E8: Essential 8
EDTA: Ethylenediaminetetraacetic acid
EHT: Engineered heart tissue
FGF: Fibroblast growth factor
FHF: First heart field
GAPDH: Glyceraldehyde-3-phosphate dehydrogenase
GATA4: GATA binding protein 4
HAND1: Heart and neural crest derivatives expressed 1
hESC: Human embryonic stem cell
hiPSC: Human induced pluripotent stem cells
hiPSC-CM: Cardiomyocyte derived from human induced pluripotent stem cell
HRA: High concentration retinoic acid
IGF-1: Insulin-like growth factor 1
IMDM: Iscove’s Modified Dulbecco’s Medium
ISL1: ISL LIM homeobox 1
LRA: Low concentration retinoic acid
LV: Left Ventricle
MEF2C: Myocyte enhancer factor 2C
MYH6: Myosin heavy chain 6
MYH7: Myosin heavy chain 7
MYL2: Myosin light chain 2
MYL7: Myosin light chain 7
NKX2.5: NK2 homeobox 5
NPPA: Natriuretic peptide A
NPPB: Natriuretic peptide B
NR2F2: Nuclear receptor subfamily 2 group F member 2
PBS: Phosphate buffer saline
PCR: Polymerase chain reaction
PFA: Paraformaldehyde
PPI: Protein-protein interaction
RA: Retinoic acid
RAR: RA receptors
RARE: Retinoic acid response elements
RIN: RNA Integrity number
RV: Right Ventricle
SEM: Standard error of the mean
SHF: Second heart field
TBX20: T-box transcription factor 20
TBX5: T-box transcription factor 5
TGF-ß1: Transforming growth factor-β1 transcription factor (TF)
t-SNE: t-distributed stochastic neighbor embedding
VEGF165: Vascular endothelial growth factor isoform 165
WGA: Wheat germ agglutinin
