# Supplementary material for: Retinoic acid modulation guides human-induced pluripotent stem cell differentiation towards left or right ventricle-like cardiomyocytes
Source: Stem Cell Res Ther. 2024 Jun 21;15:184. doi: 10.1186/s13287-024-03741-0 (PMC11191368; doi:10.1186/s13287-024-03741-0)
Supplement: Supplementary file 1 — Supplementary Material 1 [file 13287_2024_3741_MOESM1_ESM.docx]

SUPPLEMENTARY MATERIALS

**Retinoic acid modulation guides human-induced pluripotent stem cell differentiation towards left or right ventricle-like cardiomyocytes**

Hengliang Zhang^1^ ^2 6^, Payel Sen^1 2^, Jules Hamers^1 2^, Theresa Sittig^1 2^, Brent Woestenburg^1^, Alessandra Moretti^2 4 5^, Andreas Dendorfer^1 2^, Daphne Merkus^1 2 3^

^1^Walter Brendel Center for Experimental Medicine (WBex), University Clinic Munich, LMU Munich, 81377 Munich, Germany.

^2^Center for Cardiovascular Research (DZHK), Munich Heart Alliance (MHA), Partner Site Munich, 81377 Munich, Germany.

^3^Division of Experimental Cardiology, Erasmus University Medical Center, 3015 GD Rotterdam, The Netherlands.

^4^First Department of Medicine, Cardiology, Klinikum rechts der Isar, Technical University of Munich, School of Medicine and Health, Munich, Germany.

^5^Regenerative Medicine in Cardiovascular Diseases, First Department of Medicine, Klinikum rechts der Isar, Technical University of Munich, School of Medicine and Health, Munich, Germany.

^6^The First Affiliated Hospital, and College of Clinical Medicine of Henan University of Science and Technology, Luoyang, China.

Corresponding author: Daphne Merkus

Email: daphne.merkus@med.uni-muenchen.de

Table S1 50 ml EB6 medium composition

| Reagens | Concentration | Volume |
| --- | --- | --- |
| MEM Non-Essential Amino Acids (  Gibco, 11140050) | 2 % | 1 ml |
| Penicillin | 50 U/ml | 0.25 ml |
| Streptomycin | 50 µg/ml | 0.25 ml |
| Fetal Bovine Serum (PAN Biotech) | 6 % | 3 mg |
| Beta-mercaptoethanol | 0.1 mmol/L | 0.5 ml |
| DMEM/F-12 (Gibco, No. 11320033) | 90 % | 45 ml |

Table S2 50 ml EHT medium composition

| Reagens | Concentration | Volume |
| --- | --- | --- |
| B27- Insulin (Gibco, A1895601) | 2% | 1 ml |
| MEM Non-Essential Amino Acids Solution (Gibco, 11140050) | 1% | 0.5 ml |
| Penicillin | 50 U/ml | 0.25 ml |
| Streptomycin | 50 µg/ml | 0.25 ml |
| Dexamethasone (Sigma Aldrich, D4902) | 1 µmol/L | 5 µl |
| IGF-1 (PEPROTECH ,100-11) | 100 ng/ml | 10 µl |
| FGF-2 (PEPROTECH, 100-18B) | 10 ng/ml | 5 µl |
| VEGF165 (PEPROTECH, 100-20) | 5 ng/ml | 2.5 µl |
| TGF-ß1 (PEPROTECH,100-21) | 5 ng/ml | 2.5 µl |
| IMDM (Gibco, No. 12440061) | 96% | 48 ml |

IMDM: Iscove's Modified Dulbecco's Medium; DMEM/F-12: Dulbecco's Modified Eagle Medium/Nutrient Mixture F-12; EHT; engineered heart tissue; FGF-2; fibroblast growth factor-2; IGF-1: insulin-like growth factor 1; TGF-β1; transforming growth factor-β1; VEGF165: vascular endothelial growth factor 165.

Table S3 Primer list for RT-qPCR (Human)

| Gene | forward | reverse |
| --- | --- | --- |
| GAPDH | GGTCTCCTCTGACTTCAACA | AGCCAAATTCGTTGTCATAC |
| TBX5 | ACACATCGTGAAAGCAGACG | TAACTCCAGGTCATCACTGC |
| NKX2.5 | CCAAGGACCCTAGAGCCGAA | ATAGGCGGGGTAGGCGTTAT |
| MEF2C | CCAACTTCGAGATGCCAGTCT | GTCGATGTGTTACACCAGGAG |
| MYH7 | ACTGCCGAGACCGAGTATG | GCGATCCTTGAGGTTGTAGAGC |
| cTnT | AGACAGAGCGGAAAAGTGGG | TCCTTGGCCTTCTCCCTCAG |
| HAND1 | GAGAGCATTAACAGCGCATTCG | CGCAGAGTCTTGATCTTGGAGAG |
| Connexin43 | GGGACAGCGGTTGAGTCAG | TGTTACAACGAAAGGCAGACTG |
| ISL1 | ATGGGAGACATGGGAGATCCA | GCATTTGATCCCGTACAACCTGAT |
| TBX20 | TCACTGTCCGTAGTTCCGC | CCACAAATTGCTCCAGGGGT |
| CORIN | GCCGGTCTTGAGAGCTGAT | GTTCATACAGGCACCAACATAG |
| GATA4 | GCCCAGCAGGACCCC | CTATTGGGGGCAGAAGACGG |
| WNT5A | ATTCTGGCTCCACTTGTTGCT | TTCATACCTAGCGACCACCA |
| HCN4 | GGAGGCCGAGGTGCG | GGTCAGGTCCCAGTAAAATCTGA |
| ADRB1 | GACGCTCACCAACCTCTTCA | CACAGCTCGCAGAAGAAGGA |
| THBS4 | CAGCTTGGCCTAAAGGCTGT | CCGAAGATGGTGGCTGAACT |

Table S4. List of significant upregulated genes in the HRA compared to the control (Supplementary Information).

Table S5. List of significant downregulated genes in the HRA compared to the control (Supplementary Information).


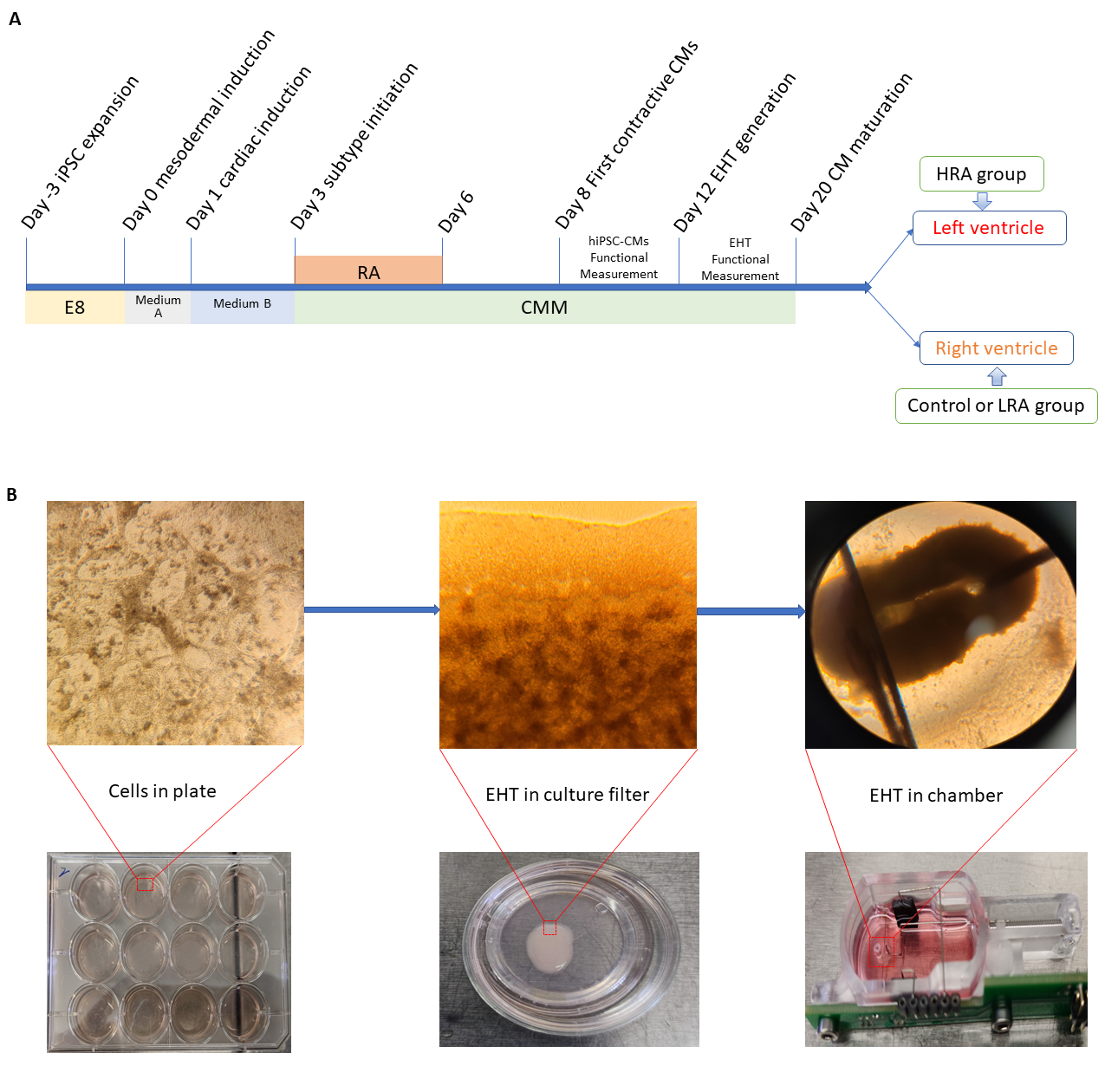


Figure S1. Detailed schematic representation of the experimental process.

A Workflow of the hiPSC-CM differentiation process.

From the 3rd to 6th days of differentiation, vehicle or RA was added to the control group (DMSO vehicle only), LRA (0.05 µM RA) and HRA (0.1 µM RA) group respectively.

B The process of making EHT and functional measurements.


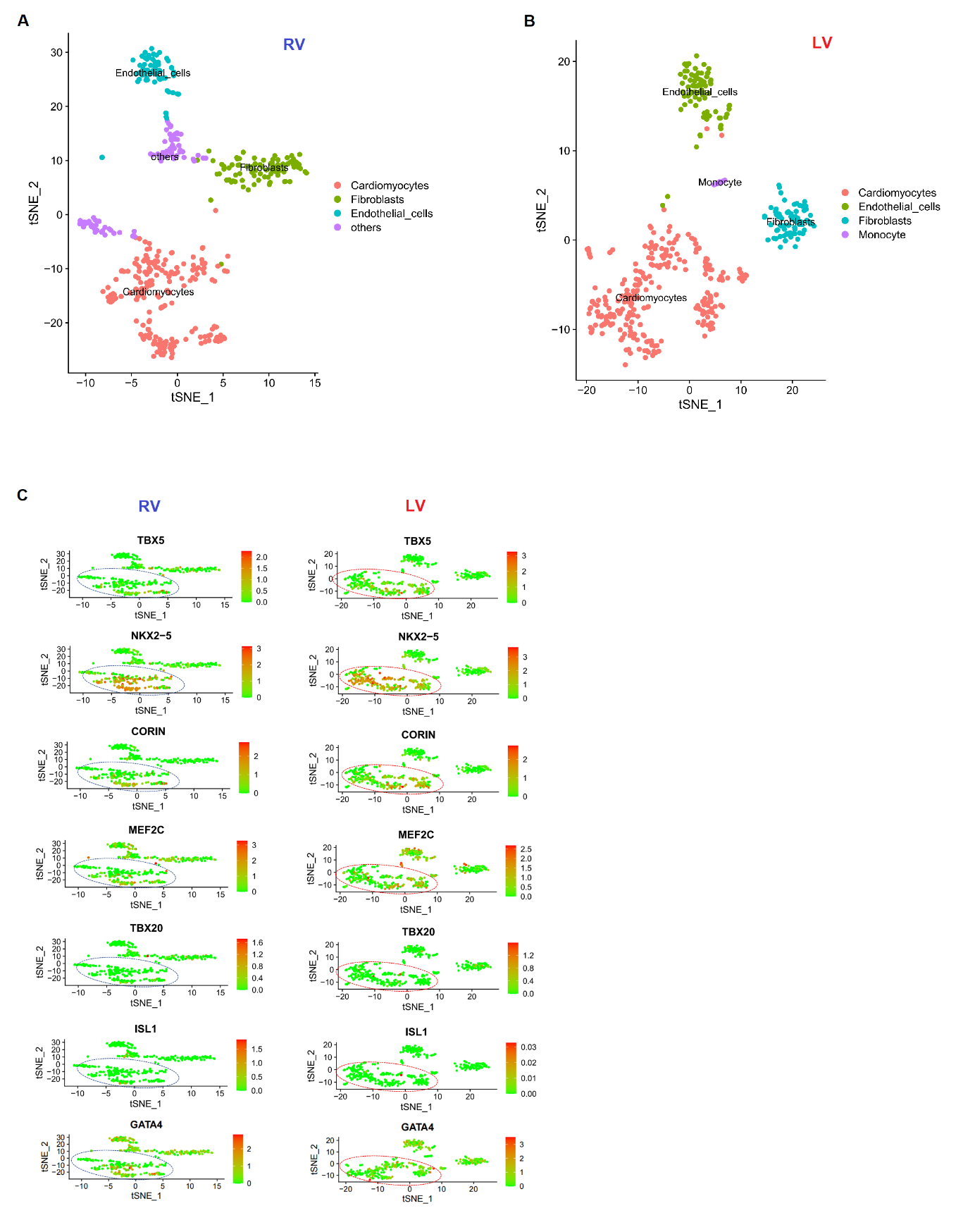


Figure S2. Transcriptome characteristics of the left and right ventricular cardiomyocytes based on analysis of the single-cell RNA sequencing dataset GSE106118, acquired from GEO.

A Spatial distribution of expression levels of transcriptome in different cell types of RV.

B Spatial distribution of expression levels of transcriptome in different cell types of LV.

C Spatial expression of marker genes in LV and RV.


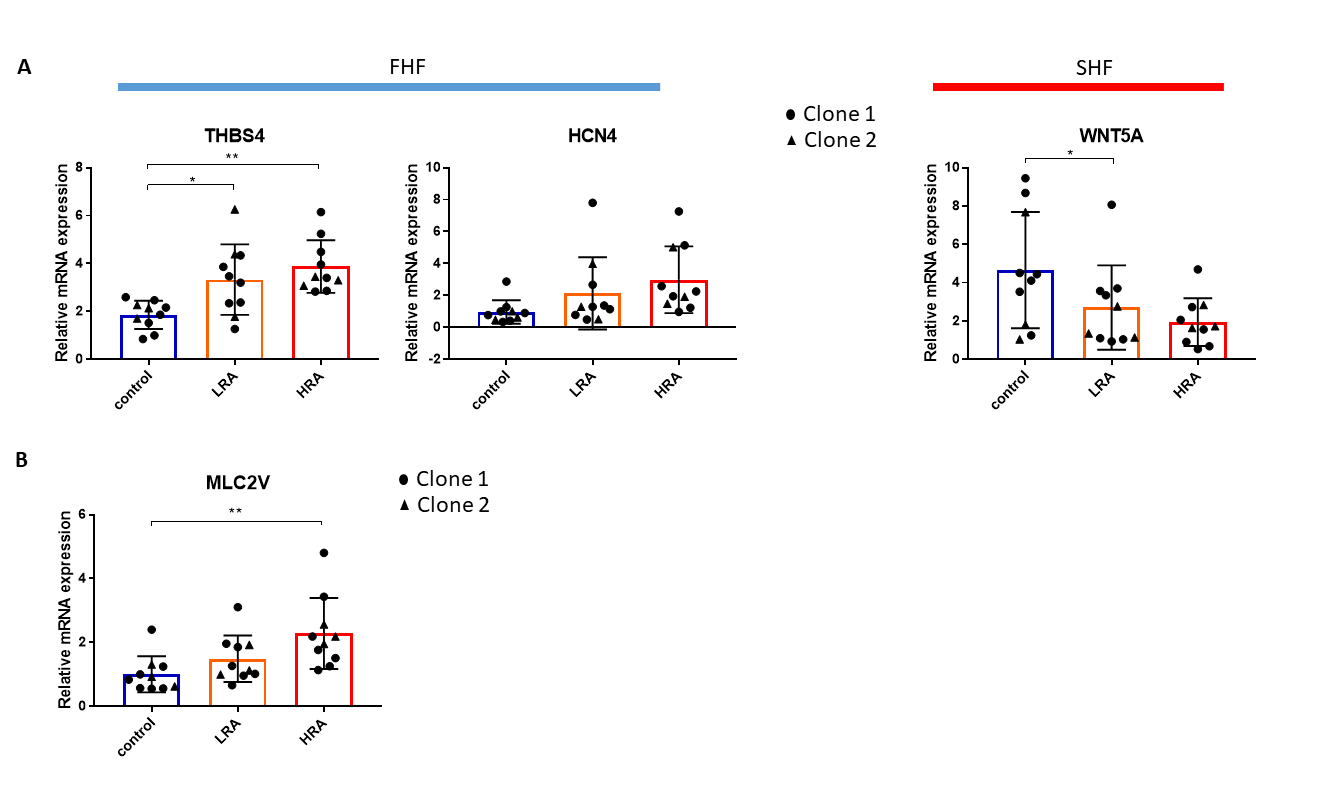


Figure S3. Marker genes expression in hiPSC-CMs.

A. FHF and SHF marker genes expression in Control, LRA and HRA-EHTs.

B. Ventricular marker genes expression in Control, LRA and HRA-EHTs.


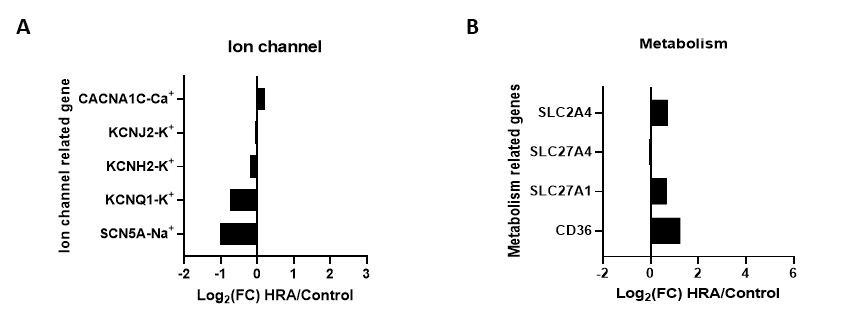


Figure S4: mRNA expression of ion channels (left) and glucose and fatty acid transporters as determined from RNA sequencing of 3 HRA-EHTs and 3 Control EHTs


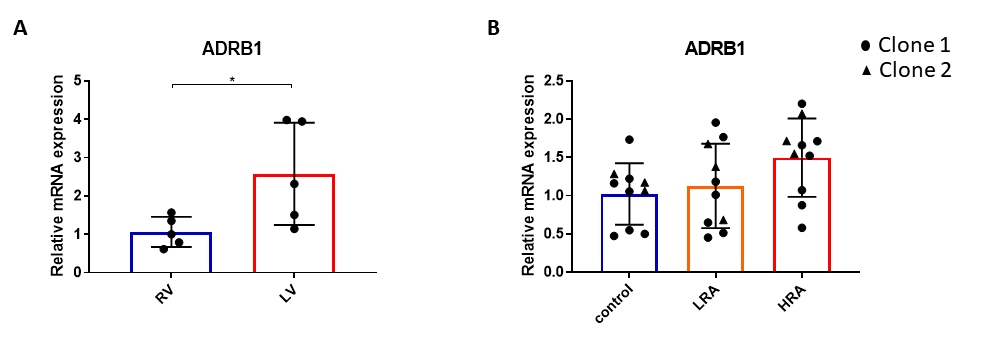


Figure S5: A: mRNA expression of ADRB1 in human adult LV and RV and in iPSC-CM exposed to different concentrations of RA.
